# Efficacy of seven and fourteen days of antibiotic treatment in uncomplicated *Staphylococcus aureus* bacteremia (SAB7): study protocol for a randomized controlled trial

**DOI:** 10.1186/s13063-019-3357-9

**Published:** 2019-05-02

**Authors:** Louise Thorlacius-Ussing, Christian Østergaard Andersen, Niels Frimodt-Møller, Inge Jenny Dahl Knudsen, Jens Lundgren, Thomas Lars Benfield

**Affiliations:** 1 Department of Infectious Diseases, Hvidovre Hospital, Copenhagen University Hospital, Kettegaard Alle 30, 2650 Hvidovre, Denmark; 2 Department of Clinical Microbiology, Hvidovre Hospital, Copenhagen University Hospital, Hvidovre, Denmark; 3 Department of Clinical Microbiology, Rigshospitalet, Copenhagen University Hospital, Copenhagen, Denmark; 4 Department of Infectious Diseases, Rigshospitalet, Copenhagen University Hospital, Copenhagen, Denmark

**Keywords:** Randomized controlled trial, *Staphylococcus aureus* bacteremia, Bloodstream infection, Treatment duration, Short-course therapy, Uncomplicated *Staphylococcus aureus* bacteremia

## Abstract

**Background:**

*Staphylococcus aureus* bacteremia (SAB) is frequently encountered in the hospital setting, and current guidelines recommend at least 14 days of antibiotic treatment for SAB in order to minimize risks of secondary deep infections and relapse. However, evidence to support these treatment recommendations remains scarce. Patients with uncomplicated SAB are known to have a low of risk of recurrence and death. Reducing treatment length in uncomplicated SAB would reduce the total consumption of antibiotics, duration of hospital admission, and potentially the risk of adverse events. With SAB7 we seek to determine if 7 days of antibiotic treatment in patients with uncomplicated SAB is non-inferior to 14 days of treatment.

**Methods/design:**

The study is designed as a randomized, non-blinded, non-inferiority, multicenter interventional study. Primary measure of outcome will be 90-day survival without clinical or microbiological failure to treatment or relapse. Secondary outcomes include the prevalence of severe adverse effects, in particular secondary infection with *Clostridium difficile*, all-cause mortality, as well as public health related costs. Patients identified with uncomplicated SAB who have received 7 days of protocol-approved antibiotics will be eligible for inclusion and randomized 1:1 in two parallel arms to either (i) discontinue antibiotic treatment at day 7 or (ii) to continue antibiotic treatment for a total of 14 days. Main exclusion criteria include signs of complicated SAB, such as the presence of secondary deep infections, persistent bacteremia, and implantable devices. Patients are followed for 6 months with clinical examinations, consecutive blood tests, and registration of adverse events. A total of 284 patients are to be included at ten centers across Denmark. The primary endpoint will be tested with a statistical non-inferiority margin of 10 percentage points.

**Discussion:**

SAB 7 will determine if 7 days of antibiotic treatment in patients with uncomplicated SAB is sufficient and safe. Results of the study will provide important knowledge on optimized SAB management and could potentially modify the current treatment recommendations.

**Trial registration:**

ClinicalTrails.gov, H-17027414. Registered on May 2, 2018. The Danish Medicines Agency (EudraCT), 2017–003529-13. Registered on October 30, 2017.

**Electronic supplementary material:**

The online version of this article (10.1186/s13063-019-3357-9) contains supplementary material, which is available to authorized users.

## Background

### Background

The extensive use of antibiotics worldwide is closely related to the increasing issues of antimicrobial resistance and antibiotic-associated infections [1, 2]. Long-course antibiotic treatment, as used for blood stream infections, plays a critical role in this context.

*Staphylococcus aureus* is one of the most frequent causes of bacterial infections in skin, bone, tissue, and blood, with bacteremia being one of the most severe presentations. In Denmark the incidence is around 25 per 100,000 population, corresponding to approximately 1800 cases of *S. aureus* bacteremia (SAB) each year [3–5]. SAB is often associated with severe complications, such as secondary deep infections, and a reported mortality rate of 20–25% [5]. However, nearly half of cases are classified as uncomplicated infections associated with a significantly lower risk of relapse and death [6, 7]. Uncomplicated SAB is partly due to in-hospital infections related to invasive procedures and the use of peripheral and central venous catheters, as well as community-acquired skin and soft tissue infections [8, 9].

Present international and Danish guidelines recommend a standard treatment regimen of a minimum of 14 days of intravenous antibiotics for SAB [6]. However, these recommendations are based on only a few randomized clinical studies and, to a greater extent, on individual expert opinion [10]. Only one minor clinical trial has evaluated the length of treatment in SAB, and this found no significant differences in outcome between 2 and 4 weeks of treatment [11]. It is known from case-based studies that 10–14 days of treatment in uncomplicated SAB are associated with few secondary complications and failure to treat events [12–15]. Interestingly, three observational studies showed that 7 days of treatment in simple catheter-associated SAB were effective and not associated with a higher risk of recurrence if the focus of the infection was eradicated. In all reported cases, follow-up blood cultures were negative and the patients showed no clinical or biochemical signs of adverse events or complications [6, 7, 16]. However, present evidence regarding length of treatment for SAB is limited and further research in the area could potentially modify current clinical practice.

### Hypothesis and aims

We hypothesize that 7 days of antibiotic treatment in uncomplicated SAB is non-inferior to 14 days of treatment.

We primarily seek to compare 7 and 14 days of antibiotic treatment in patients with uncomplicated SAB in terms of mortality and the prevalence of microbiological and clinical failure to treat and recurrence within 90 days of diagnosis.

The study aims to explore the possibility of reducing the consumption of antibiotics, as well as shortened hospital admission, and thereby potentially decrease the risk of adverse events and microbial resistance development.

### Primary and secondary outcomes

#### Primary outcome

- The primary outcome is 90-day survival without clinical or microbiological failure to treatment or relapse in patients treated with 7 days versus 14 days of antibiotic therapy

#### Secondary outcomes

- All-cause mortality on days 14, 28, 90, and 180
- Microbiological failure to treatment
- Microbiological relapse
- Clinical failure to treatment
- Desirability of outcome ranking (DOOR)
- Hospital admissions during follow-up
- Severe adverse events grade ≥ 3
- Acute renal injury
- *Clostridium difficile* infection
- Infection with multidrug-resistant organisms
- Health-associated costs associated the treatment lengths

## Methods/design

### Design and randomization

The study is a randomized, non-blinded, non-inferiority, interventional study. Confirmation of study eligibility will be performed by entering key variables into a web-based program (REDCap) with subsequent automatic patient randomization into two parallel arms (ratio 1:1): treatment regimens of 7 or 14 days, respectively. Randomization lists will be generated centrally in random blocs and stratified according to catheter-associated infections as well as center/hospital. For a detailed description of the items included in the SAB7 study protocol, please see the SPIRIT table in Additional file 1.

### Ethics and regulatory considerations

The study has been approved by the relevant regulatory authorities, including The Danish Medicines Agency (EudraCT 2017–003529-13), Research Ethics Committee of the Capital Region of Denmark (H-17027414), and The Danish Data Protection Registry (ID AHH-2017-086, I-Suite no.: 05891). The study will be conducted according to ICH-GCP (guidelines for Good Clinical Practice (GCP)) and monitored by GCP units in Denmark. Informed consent is obtained from all study participants.

### Setting

The following ten hospitals, representing all five regions of Denmark, will be invited to participate in the study: Hvidovre Hospital, Rigshospitalet, Herlev Hospital, Hillerød Hospital, Roskilde Hospital, Odense University Hospital, Kolding Hospital, University Hospital of Aarhus, Herning Hospital, and University Hospital of Aalborg.

### Study population

**Fig. 1:**
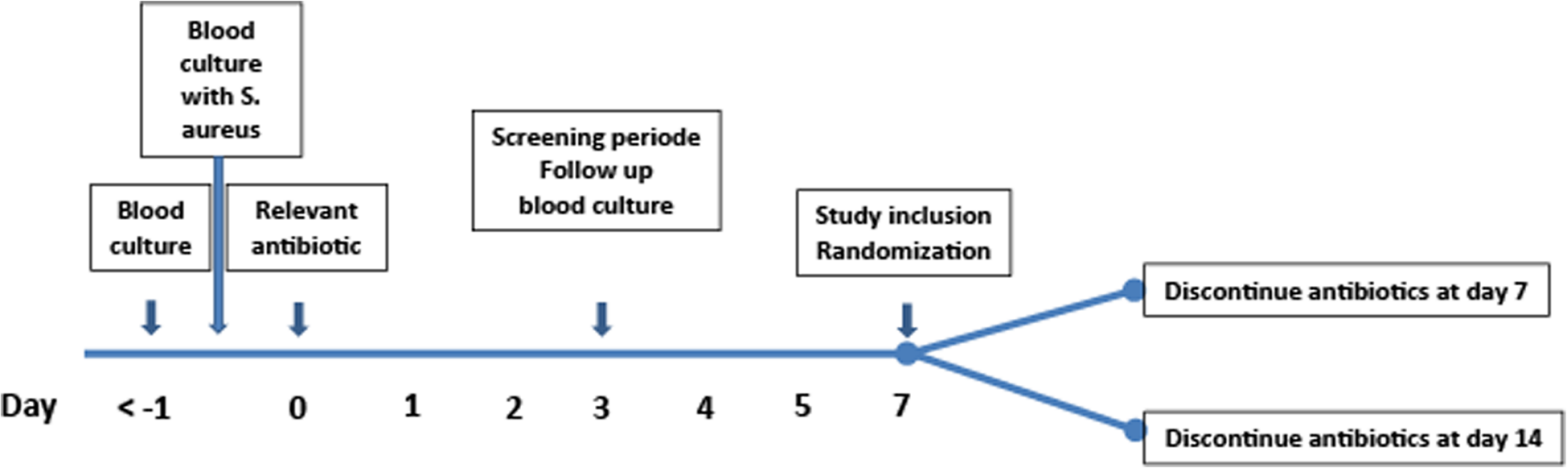
Timeline of SAB7

Uncomplicated SAB is defined according to the guidelines of the Infectious Disease Society of America [17]. Briefly, patients are required to be free of signs of infectious endocarditis and have a negative follow-up blood culture, a physical examination without signs of metastatic foci, absence of fever 48–72 h after initiation of appropriate antibiotic therapy, and no implantable devices. Prosthetics in joint and bones implanted > 6 months prior to SAB will be accepted if the patients show no clinical signs of infection involving the prosthetics. Inclusion and exclusion criteria are listed in detail in Table 1. Eligible patients must fulfill all of the inclusion and none of the exclusion criteria (Fig. 1). Cases are identified through the respective departments of microbiology and infectious diseases at each center. All participants are hospitalized at enrollment.

**Table 1:** Inclusion and exclusion criteria

Inclusion criteria	Exclusion criteria
• Age > 18 years	• Persistence of *S. aureus* bacteremia before randomization (*S. aureus* positive follow-up blood culture obtained within 48–120 h of the first positive blood culture)
• Blood culture positive for *Staphylococcus aureus*	• Polymicrobial infection
• Antibiotic treatment with antimicrobial activity to *S. aureus* administrated within 12 h of the first positive blood culture	• Antibiotic treatment with no antimicrobial activity to *S. aureus* administered more than 12 h after the first positive blood culture
• Temperature < 37,5 °C at randomization	• Endocarditis or other intracardiac infection demonstrated with transthoracic or transesophageal echocardiography
*• S. aureus* negative follow-up blood culture obtained 48–120 h after microbiologically verified SAB	• Previous history of endocarditis
• Patients written consent obtained	• Pacemaker or other intracardiac implant
	• Failure to remove a likely focus of infection, such as central venous catheter, within 72 h of the first positive blood culture
	• Vascular grafts
	• Pneumonia or infection involving bone, joints, or prosthetics
	• Previous bone/joint infection
	• *S. aureus* infection within the last 90 days
	• Pregnancy or breastfeeding
	• Neutropenia (blood neutrophils < 1.0 × 10^9^/l)
	• Untreated cancer
	• Chemotherapy within 90 days.

### Treatment

Patients will receive antibiotic treatment adhering to local and national guidelines as well as according to the susceptibility of the respective isolate. Participation in the study will only affect treatment duration and will have no influence on the choice of treatment with respect to type and dose of antibiotic agent. Patients entering the study have already received 7 days of antibiotic treatment. According to randomization, patients will (i) discontinue antibiotic treatment at day 7 or (ii) continue antibiotic treatment for a total of 14 days. Antibiotics considered appropriate for empiric treatment of SAB are listed in Table 2.

**Table 2:** Acceptable empiric antibiotic treatment of SAB

Antibiotic	Form * (oral or iv)	Standard dose *	Frequency *	Dose adjustment *
Piperacillin + tazobactam (MSSA)	IV	4 g + 0.5 g	Every 8 h	Renal impairment
Dicloxacillin (MSSA)	Oral or IV	1 g	Every 6 h	Weight
Flucloxacillin (MSSA)	Oral or IV	1 g	Every 6–8 h	Renal impairment
Cloxacillin (MSSA)	Oral or IV	1 g	Every 6–8 h	Renal impairment
Cefuroxim (MSSA)	IV	750 mg 1.5 g	Every 6 h Every 8 h	Renal impairment
Clindamycin (MSSA + MRSA)	Oral	600 mg	Every 6–8 h	–
Macrolides (MSSA)				
Claritromycin	Oral or IV	500 mg	Every 12 h	Renal and liver impairment
Vancomycin (MSSA + MRSA)	IV	1 g IV	Every 12 h	Renal impairment Se- vanco
Linezolid (MRSA + MSSA)	Oral or IV	600 mg	Every 12 h	–
Rifampicin (MSSA)	Oral	300–600 mg	Every 8 h	–
Meropenem (MSSA)	IV	1–2 g	Every 8 h	Renal impairment
Moxifloxacin (MSSA)	Oral or IV	400 mg	Every 24 h	–
Aminoglycosides (MSSA)				
-Gentamycin	IV	5 mg/kg	Every 24 h	Renal impairment

*Standard recommendations

*IV* intravenous, *MSSA* methicillin-sensitive *Staphylococcus aureus*, *MRSA* methicillin-resistant *Staphylococcus aureus*, * Se-vanco* serum-vancomycin

### Assessment and data collection

**Fig. 2:**
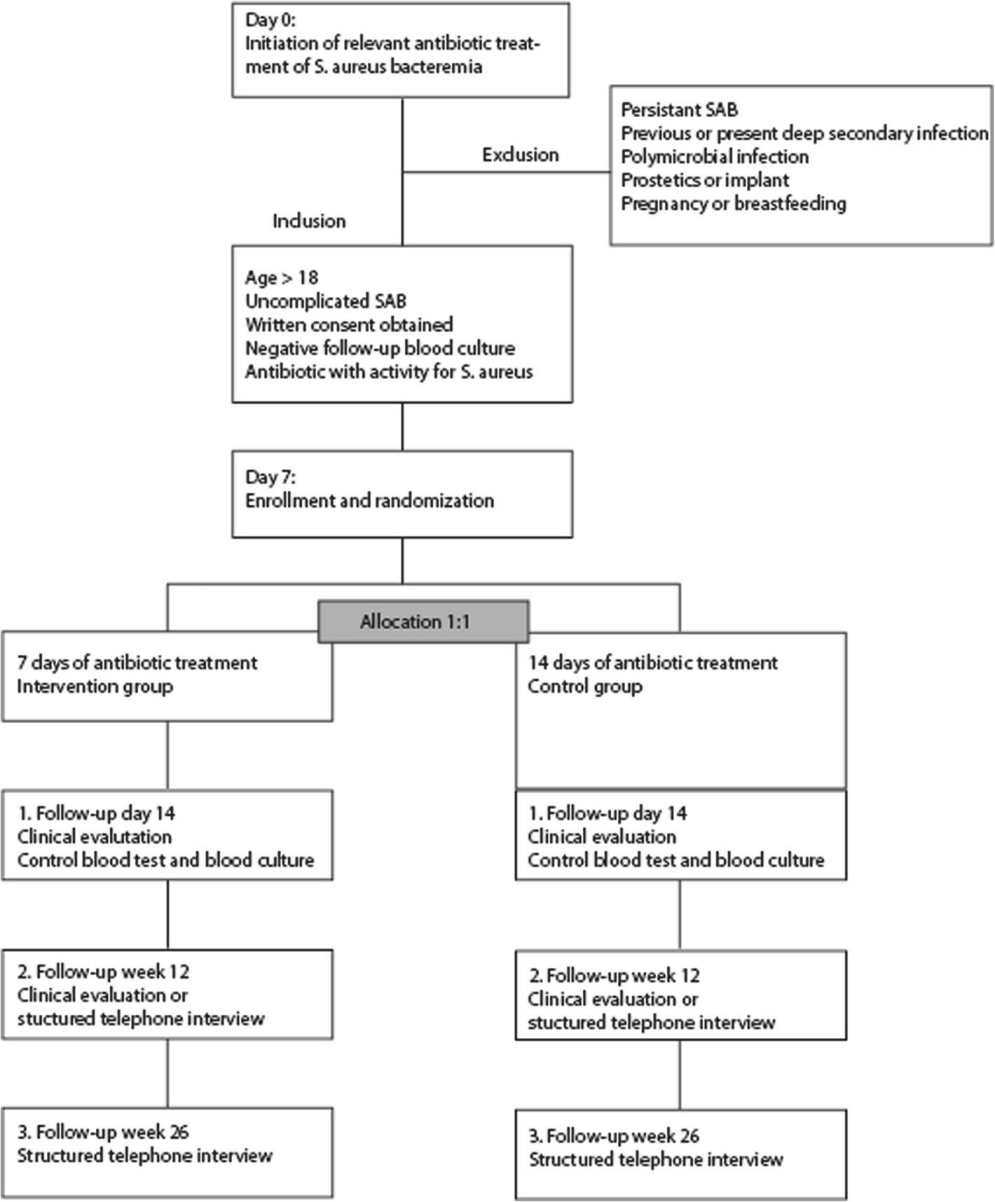
Flowchart of SAB7

**Fig. 3:**
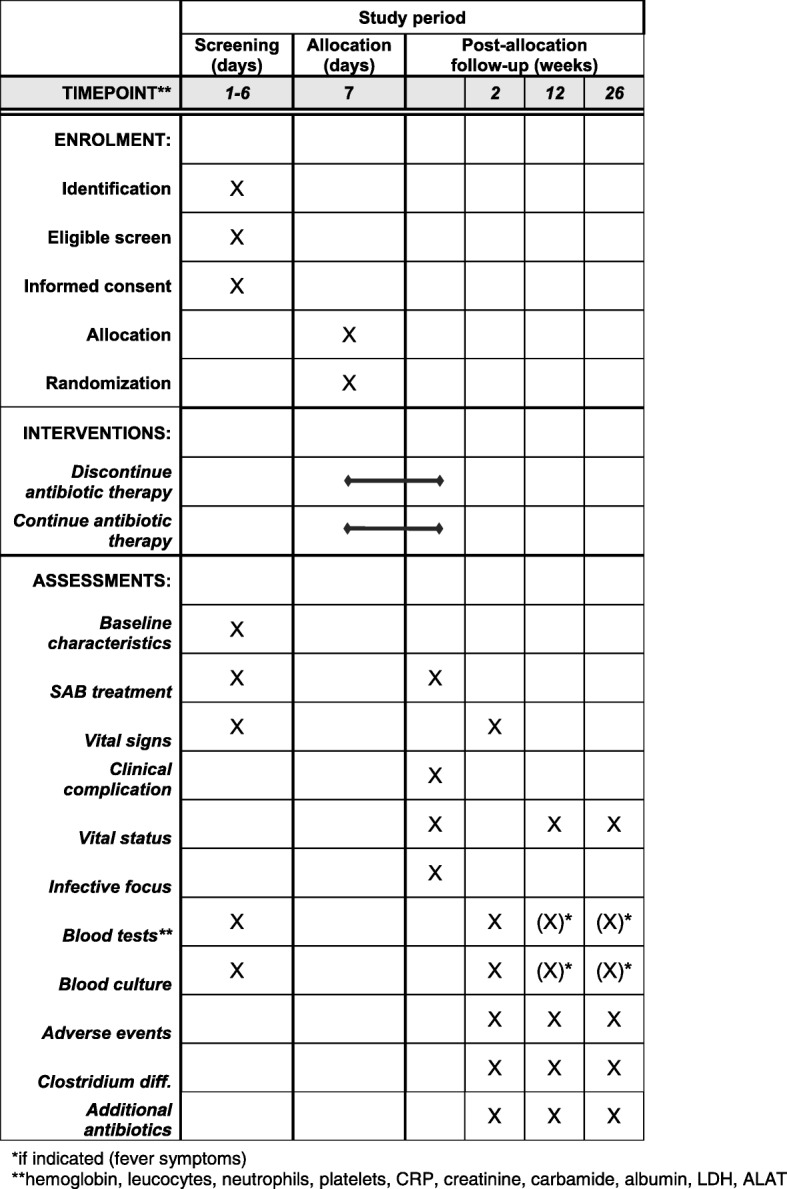
Data collection and follow-up for patients enrolled in SAB7

A summary of collected data is presented in Fig. 3. Study participants will be followed by doctors at the respective centers for 6 months after the SAB diagnosis (Figs. 2 and 3). Hospitalized patients will be assessed in accordance with the study protocol and local hospital standards. Day 0 is defined as the initiation of antibiotic therapy with antimicrobial effects against SAB (Fig. 1). Days 1–6 serve as a screening period to determine study eligibility. Eligible patients will be included in the trial and randomized at day 7. To evaluate outcome efficacy patients will undergo three follow-up examinations on day 14 and weeks 12 and 26, respectively. The first follow-up visit will consist of a physical examination, whereas the second and third follow-up visits will consist of a clinical consultation or a standardized telephone interview (Fig. 2). In addition to the scheduled follow-up visits, patients will at discharge be thoroughly instructed on contacting the treating physician if they develop symptoms or fever at any time between follow-up visits. Any signs of fever must lead to a clinical evaluation, blood testing, and blood culture. Follow-up data and laboratory tests are described in Fig. 3. Additional follow-up regarding comorbidity and other clinical variables is conducted by the use of hospital and national databases. All data are registered in an electronic case report form.

### Outcome measures

#### Primary outcome

Data regarding the composite primary endpoint, 90-day survival without clinical or microbiological failure to treatment or relapse, will be acquired through patient interviews, clinical consultation, and microbiological tests as well as hospital and national administrative registries.

Microbiological failure to treatment is defined as verified *S. aureus*, of the same genotype (based on *spa* type) as the initial infection, less than 7 days after treatment termination.

Microbiological SAB relapse is defined as demonstration of the same *S. aureus* genotype as the initial infection more than 7 days after treatment termination, demonstrated by molecular-based typing [18, 19]. Verification of *S. aureus* as the infectious agent in question will be performed by microbiological analysis of biological material such as blood, synovial fluid, etc.

Clinical failure to treatment or relapse is defined as initiation of anti-staphylococcal therapy for more than 48 h on suspected clinical recurrence.

Data on vital status during follow-up are obtained from local hospital registries and the Danish Civil Registration System.

Data regarding the primary endpoint will be evaluated and determined by an independent committee (Endpoint Review Committee) blinded to randomization.

#### Secondary outcome

The components of the composite primary outcome will also be assessed separately as a secondary outcome, which is defined as described above. Additional secondary objectives are overall survival at days 14, 28, and 180 and the presence of severe adverse events, including the prevalence of *C. difficile*-associated diarrhea, infection with a multidrug-resistant organism and grade 3 or above adverse events as defined elsewhere (https://ctep.cancer.gov/protocoldevelopment/electronic_applications/ctc.htm). The prevalence of adverse events will be derived from patient interviews and laboratory reports, including, if relevant, documentation of the microbiological method applied for verification of *C. difficile*. Additionally, follow-up blood tests will also be used to assess adverse events by screening for possible antibiotic-related affects on, e.g., liver and kidney function. Acute kidney injury is defined as a 1.5-fold increase in plasma creatinine or a 25% decrease of estimated glomerular filtration rate (GFR).

Our definition of multidrug-resistant organisms will rely on identification of resistant bacteria in a clinical specimen obtained only from a patient with a clinical infection. As such, routine screening of asymptomatic individuals to identify potential colonization with a resistant microorganism will not be performed.

To exclude potential effects of concomitant medical therapy not related to SAB treatment, additional antibiotic consumption within the study period is registered.

Public health-related costs will be estimated from a general consideration of the expenses associated with hospitalization for SAB.

## Statistics

### Pilot phase

Based on local and national databases, we expect a failure to treat and relapse rate of < 5% for patients with uncomplicated SAB alive at day 7 and a 90-day all-cause mortality rate of approximately 7%, corresponding to a recovery rate of 88% [20]. However, available data on recurrence and death in this select group of patients is sparse. Consequently, an adaptive trial design is used in which the event rate is reassessed and the sample size and the non-inferiority margin adjusted if appropriate. The reassessment will occur at the second interim analysis when 100 patients have entered the study and completed 90 days of follow-up. Reassessment after the second interim analysis may lead to one of four changes to the study: 1) sample size and non-inferiority margin will remain unchanged because the event rate is 12%; 2) sample size and non-inferiority margin will be lowered because the event rates is < 12%; 3) sample size will be increased because the event rate is > 12% while the non-inferiority margin will remain unchanged; or 4) the trial will be terminated due to safety concerns.

The regulatory authorities will be informed of the result of the pilot phase and interim analysis and potential adjustments to sample size.

### Sample size

Non-inferiority is defined as a difference in primary endpoint of up to 10%. Assuming a treatment response of 88%, statistical power of 80%, a statistical significance level of 5% and a 2% loss to follow-up rate, 284 randomized patients are require in order to exclude predefined difference in the two groups. Sample size was estimated using simulation, with the assumption of a 12% failure in both groups and a non-inferiority margin of 10%.

### Interim analysis

We will perform two interim analyses when 30 and 100 patients have completed the study, respectively. This serves to evaluate the primary and secondary endpoints and potential adverse events by an independent data and safety monitoring board (DSMB).

The Haybittle-Peto method is applied to demonstrate overwhelming differences between the two treatment groups that necessitate premature termination of the trail. A significant *p* value of 0.001 in the interim analyses will correspond to a *p* value of 0.05 in the final analysis.

## Discussion

SAB represents a persistent challenge in bacterial infections. Research in this field has until now mainly focused on identifying risk factors associated with secondary complications, handling the increasing frequency of methicillin resistant *Staphylococcus aureus* (MRSA) infections, and, to a lesser extent, improving the present treatment strategies. The SABATO trail is currently investigating if early oral switch therapy is safe and effective for patients with low-risk SAB [21]. Even though this study may address issues with long-course intravenous treatment, results will not affect the overall consumption of antibiotics nor treatment length. To the best of our knowledge, this is the first randomized clinical study to assess reduced treatment length in SAB and the efficacy of 7 days of treatment. We chose SAB-associated death, microbiological and clinical failure to treat, and recurrence as the primary endpoint, as we feel this is the most accurate measure of the effectiveness of the treatment.

SAB7 will, regardless of the outcome, provide important information on optimized treatment of patients with uncomplicated SAB. At best, the study will provide evidence that a reduction in treatment length in uncomplicated SAB is sufficient and safe. If so, this may lead to a paradigm shift in the treatment of patients with uncomplicated SAB. Results of SAB7 will potentially improve future treatment in several ways. Reducing treatment length in uncomplicated SAB could induce earlier discharge from the hospital, decreased risk of hospital-acquired infections, decreased risk of severe adverse events, and an increased quality of life. Additionally, from a national and international perspective, decreasing the consumption of antibiotics will play an important role in preventing resistance development and secure future antimicrobial treatment.

## Trial status

The first patient was recruited on July 20, 2018. Recruitment is expected to be completed in May 2021.

## Additional file

Additional file 1: SPIRIT 2013 checklist. (DOC 122 kb)

## Abbreviations

ALAT: Alanine amino transferase
CRP: C-reactive protein
DSMB: Data and Safety Monitoring Board
GCP: Good clinical practice
ICH: International Conference of Harmonization
LDH: Lactate dehydrogenase
MRSA: Methicillin resistant *Staphylococcus aureus*
MSSA: Methicillin sensitive *Staphylococcus aureus*
SAB: *Staphylococcus aureus* bacteremia
